# Solid Oxide Cells with Phase-Inversion Tape-Casted Hydrogen Electrode and SrSc_0.175_Nb_0.025_Co_0.8_O_3−δ_ Oxygen Electrode for High-Performance Reversible Power Generation and Hydrogen Production

**DOI:** 10.3390/molecules27238396

**Published:** 2022-12-01

**Authors:** Meiting Yang, Changjiang Yang, Mingzhuang Liang, Guangming Yang, Ran Ran, Wei Zhou, Zongping Shao

**Affiliations:** 1 State Key Laboratory of Materials-Oriented Chemical Engineering, College of Chemical Engineering, Nanjing Tech University, Nanjing 211816, China; 2 WA School of Mines: Minerals, Energy and Chemical Engineering, Curtin University, Perth, WA 6845, Australia

**Keywords:** solid oxide ells, hydrogen electrode, phase-inversion tape-casting, oxygen electrode, SrSc_0.175_Nb_0.025_Co_0.8_O_3−δ_

## Abstract

Solid oxide cells (SOCs) have been considered as a promising energy conversion and storage device. However, state-of-the-art cells’ practical application with conventionally fabricated Ni-(Y_2_O_3_)_0.08_(ZrO_2_)_0.92_ (YSZ) cermet hydrogen electrode and La_0.8_Sr_0.2_MnO_3_ perovskite oxygen electrode is strongly limited by the unsatisfactory performance. Instead, new advances in cell materials and fabrication techniques that can lead to significant performance enhancements are urgently demanded. Here, we report a high-performance reversible SOC that consisted of a combination of SrSc_0.175_Nb_0.025_Co_0.8_O_3−δ_ (SSNC) and phase-inversion tape-casted Ni-YSZ, which served as the oxygen and hydrogen electrode, respectively. The hydrogen electrode synthesized from phase-inversion tape-casting showed a high porosity of 60.8%, providing sufficient active sites for hydrogen oxidation in the solid oxide fuel cell (SOFC) mode and H_2_O electrolysis in the solid oxide electrolysis cell (SOEC) mode. Accordingly, it was observed that the maximum power density of 2.3 W cm^−2^ was attained at 750 °C in SOFC mode and a current density of −1.59 A cm^−2^ was obtained at 1.3 V in SOEC mode. Hence, these results reveal that the simultaneous optimization of oxygen and hydrogen electrodes is a pragmatic strategy that improves the performance of SOCs, which may significantly accelerate the commercialization of such an attractive technology.

## 1. Introduction

With the rapid consumption of fossil fuels and aggravation of the global climate crisis, innovative technologies must be developed to utilize renewable energies such as solar and wind power effectively. Considering the intermittent nature of renewable energy, energy storage technologies are needed to achieve high-quality renewable energy use. Hydrogen energy has attracted widespread attention as an energy carrier due to having high calorific value and no pollution [1,2,3]. In the hydrogen production process, using a solid oxide electrolysis cell (SOEC) for water electrolysis has been extensively studied owing to its high energy efficiency and ease of product separation and modular operation [4,5,6,7]. Noteworthily, solid oxide cells (SOCs) can operate in two modes, regarded as SOEC and solid oxide fuel cell (SOFC), meaning that energy can be efficiently stored and regenerated by alternately switching between the different modes [8,9,10]. The integration of hydrogen production, SOEC technology utilizing renewable energy, and SOFC technology generating power from hydrogen will provide a sustainable renewable energy-based clean energy system for the future.

Recently, many researchers have discovered that the oxygen electrode plays a critical role in developing high-performance and stable SOCs [11,12,13,14]. In the SOFC mode, the oxygen electrode is used as an electrocatalyst for an oxygen reduction reaction (ORR), whereas it acts as an oxygen evolution reaction (OER) catalyst in the SOEC mode. An ideal oxygen electrode for SOCs should have good activity and durability for both the ORRs and OERs. Cobalt-based perovskite oxides such as La_0.6_Sr_0.4_Co_0.2_Fe_0.8_O_3−δ_ (LSCF) are the most intensively investigated oxygen electrodes in SOCs [12,15]. Unfortunately, these materials have not exhibited excellent ORR activity in the SOFC mode, especially at low to intermediate temperatures. Moreover, it was found that the effects of water vapor and chromium poisoning cannot be negligible in terms of the electrochemical performance of the LSCF electrode in the SOEC mode, resulting in rapid performance degradation [16,17]. Many works have reported that oxygen electrode performance is related to many factors, such as surface area, oxygen vacancy concentration, and oxygen transport rates. Several perovskite oxides derived from the SrCoO_3−δ_ parent oxide, such as Ba_0.5_Sr_0.5_Co_0.8_Fe_0.2_O_3−δ_ (BSCF) and BaCo_0.4_Fe_0.4_Zr_0.1_Y_0.1_O_3−δ_ (BCFZY), have showed superior ORR activity under intermediate temperatures [18,19,20,21]; for instance, this result was observed that the BSCF electrode has a lower area-specific resistance (ASR) of 0.1 Ω cm^2^ when working at 600 °C in the SOFC mode and at a current density of −0.45 A cm^−2^ at 850 °C in the SOEC mode [18,19]. Recently, Sc and Nb co-doped perovskite oxide denominated SrSc_0.175_Nb_0.025_Co_0.8_O_3−δ_ (SSNC) was demonstrated to be an outstanding electrode for the SOFC mode. Moreover, the single cells fabricated by SSNC can achieve a maximum power density of 910 mW cm^−2^ at 500 °C with hydrogen fuel [22]. However, the OER performance of SSNC in the SOEC mode has not been further studied.

Apart from the oxygen electrode, there is another type of excellent electrocatalyst, the hydrogen electrode, that plays a crucial role concerning both hydrogen electro-oxidation in the SOFC mode and water splitting in the SOEC mode. Recently, considerable attention has also been paid to the development of a practical hydrogen electrode for SOCs [23,24,25,26,27]. Ni-(Y_2_O_3_)_0.08_(ZrO_2_)_0.92_ (YSZ) composite is the state-of-the-art anode for the SOFC mode due to its favorable catalytic activity for hydrogen electro-oxidation, high conductivity, good thermomechanical compatibility with the YSZ electrolyte, and relatively low cost, which is also widely used in SOCs. However, the hydrogen electrode prepared by the conventional tape-casting method usually showed low porosity, thus introducing massive polarization resistance in the SOEC mode, especially under a high polarization current. This suggests that optimizing the porosity of electrode may contribute to increase the SOC’s performance. Some studies have applied a phase-inversion tape-casting technique with NMP as a solvent, PESF as a binder, and PVP as a dispersant to improve the morphology and porosity of the Ni-based hydrogen electrode [28,29,30,31].

In this study, we conducted the simultaneous improvement of both oxygen and hydrogen electrodes to achieve a high performance of SOCs. It was demonstrated that SSNC is an excellent cathode for SOFC, and for the first time, we proved that SSNC is also a suitable oxygen electrode in the SOC mode; thus, it was adopted as the oxygen electrode in the SOCs. We also report a Ni-YSZ hydrogen electrode with a honeycomb-like structure synthesized by a novel phase-inversion tape-casting method and having a much-increased electrode porosity. Combining the SSNC oxygen electrode and Ni-YSZ hydrogen electrode casted by phase-inversion tape has a remarkable performance in both two modes: a maximum power density of 2.3 W cm^−2^ at 750 °C in the SOFC mode and a current density of −1.59 A cm^−2^ at 1.3 V in the SOEC mode, respectively.

## 2. Results and Discussion

### 2.1. Microstructure of Hydrogen Electrode

It was reported that the hydrogen electrode’s porosity had a considerable influence in terms of the electrode performance, and a highly porous electrode microstructure could result in an improvement of the electrochemical performance of single cells [32,33,34,35]. Here, to emphasize the importance of microstructure optimization regarding the performance of hydrogen electrodes in SOFCs, two types of Ni-YSZ hydrogen electrodes with different structures were fabricated by the tape-casting and phase-inversion tape-casting methods, respectively.

**Figure 1:**
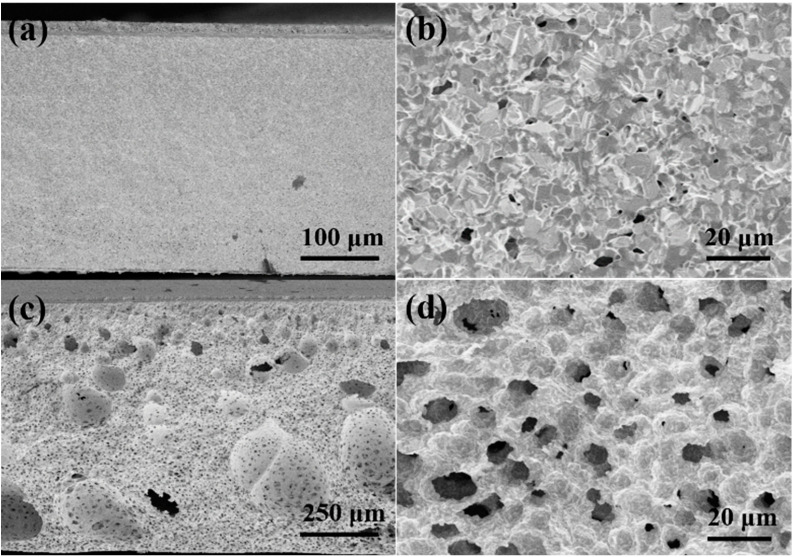
Cross-sectional FE-SEM images of hydrogen electrodes produced by tape-casting (**a**,**b**) and phase-inversion tape-casting (**c**,**d**) after calcining at 1400 °C for 5 h (not reduced).

The cross-sectional FE-SEM images of two hydrogen electrodes calcined at 1400 °C for 5 h before tests are shown in Figure 1a,b for the tape-casted electrode and Figure 1c,d for the electrode fabricated from the phase-inversion tape-casted electrode, respectively. Obviously, the hydrogen electrode produced by the conventional tape-casting method showed poor porosity after calcining at 1400 °C, and there are only a few 1–2 μm tortuous and irregular pores (Figure 1a,b). The porosity of this electrode after the reduction would be mainly attributed to the contraction due to the reduction from NiO to Ni. However, for the electrode prepared from the phase-inversion tape-casting method, in addition to some unevenly distributed large pores (30–250 μm), there are also many uniformly distributed small pores (~10 μm), which takes after the honeycomb structure (Figure 1c,d). Such honeycomb-type pores were not observed in the electrodes prepared by others based on the similar phase-inversion tape-casting method. Such a difference could result from the different casting solvents used in the phase-inversion tape-casting processes. Many researchers have obtained finger-like macroporous structures by using *N*-Methyl pyrrolidone (NMP) as a solvent [28,29,30,31]. It is known that NMP is an oily solvent, which slowly exchanges with water during the phase-inversion process and causes finger-like macropore formations. In this study, ethanol and xylene were used as the solvents. It is well-known that ethanol is readily miscible with water; thus, finger-like macropores did not appear. Meanwhile, due to xylene’s presence, a regular and ordered honeycomb structure can be formed if H_2_O entered the casting tape (Figure S1). Such a conclusion was further verified by thermogravimetric-mass spectrometric (TG-MS) testing of the casted tapes prepared by two different methods; the results are shown in Figure S2. Within the temperature range of 50–1000 °C, the calculated weight losses of the conventional casted tape and the phase-inversion casted tape were 16.2% and 14.7%, respectively (Figure S2a). In the same test interval, the conventional casted tape released slightly more water than prepared by the phase-inversion method, which was mainly caused by the immersion of the casted tape in water and not being thoroughly dried. However, the conventional casted tape released significantly more CO_2_ (Figure S2b) because the ethanol was entirely dissolved in the H_2_O, and part of the xylene was exchanged with H_2_O during the phase inversion process. Jin et al. also used glycol as a non-solvent and NMP as a solvent to obtain honeycomb-like pores in the electrode by the phase-inversion method; unfortunately, these honeycomb structures were destroyed after the electrochemical test [36]. Hence, there is no doubt that the ordered honeycomb-like structure of the hydrogen electrode would benefit the mass transfer of fuels and by-products formation in the operation of SOCs [34].

The surface morphology of the two hydrogen electrodes was also examined with the results shown in Figure S3. Unlike the dense hydrogen electrode prepared by the tape-casting method (Figure S3a,b), there were many pores with a size of ~5 μm on the surface of the hydrogen electrode generated by the phase-inversion tape-casting method (Figure S3c,d). Such rich pores facilitated the modification of the hydrogen electrode using an impregnation method. The electrode should usually be pre-reduced to introduce sufficient pores before conducting the solution-impregnated modification to modify the electrode through impregnation [37,38]. Due to the rich pores, the hydrogen electrode made by the phase-inversion tape-casting method can be directly applied to the surface modification through solution impregnation, thus greatly simplifying the preparation process and effectively reducing the fabrication cost. To verify the wettability of the surface on different hydrogen electrodes, a drop of water was dropped on the different hydrogen electrodes surface simultaneously, and the images were taken after 3 s (Figure S4). Obviously, the water on the surface of the phase-inversion tape-casting hydrogen electrode was wholly impregnated into the electrode, while that on the surface of the conventional tape-casting hydrogen electrode was hardly absorbed due to the low porosity.

**Figure 2:**
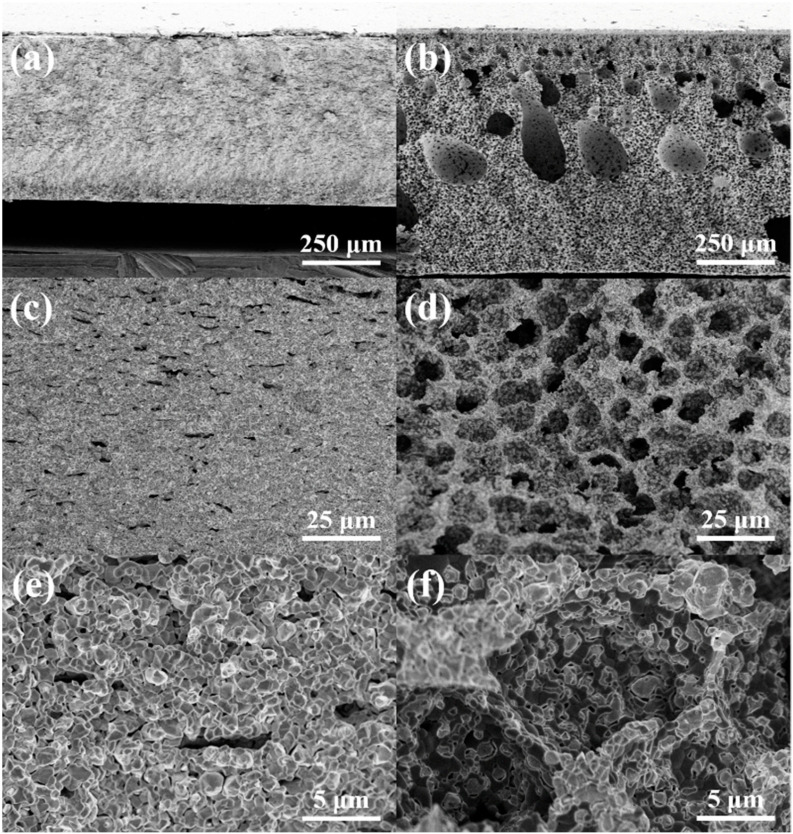
The cross-sectional FE-SEM images of tape-casting (**a**,**c**,**e**), and phase-inversion tape-casting (**b**,**d**,**f**) hydrogen electrodes after the cell test (reduced).

The cross-sectional FE-SEM images of the two different hydrogen electrodes fabricated by the traditional tape-casting and phase-inversion tape-casting methods after the cell test (reduced) were also conducted. Under the cast slurry with the same thickness, the hydrogen electrodes’ thickness increased from the initial ~500 μm to ~750 μm before and after the phase inversion (Figure 2a,b). The variation in the thickness of the hydrogen electrode also proves that the phase-inversion tape-casting method is a remarkably simple and effective method for improving electrode porosity. Because NiO was usually reduced to Ni under a reducing atmosphere, the hydrogen electrode produced by the conventional tape-casting method also clearly demonstrated a connected porous structure (Figure 2c,e), and there were also small micropores between the NiO and YSZ particles. Except for the micropores in the space of different Ni and YSZ particles, the hydrogen electrode prepared by the phase-inversion method also shows sufficient ordered honeycomb-structure pores with a size of ~10 μm (Figure 2d,f). Therefore, the hydrogen electrode prepared by the phase inversion method demonstrated a better continuous transitional microstructure, which will facilitate fuel and product transportation and electrode electrochemical reactions.

**Figure 3:**
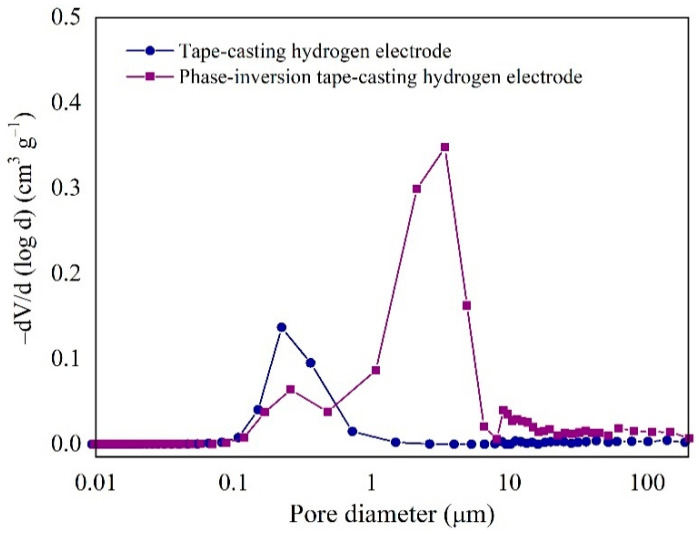
Pore size distribution of tape-casting and phase-inversion tape-casting hydrogen electrodes calcined at 1350 °C after reduction under hydrogen atmosphere at 750 °C for 5 h.

The pore size distribution of Ni-YSZ substrate was evaluated through mercury porosimetry measurement, with the results shown in Figure 3. Only one primary pore size was detected in the electrode prepared from the conventional tape casting method, i.e., pore size of 0.1–1 μm in diameter, and such pores were mainly attributed to the void space between the Ni and YSZ particles. However, there are two domains of pores in the electrode synthesized by the phase-inversion tape-casting method: one corresponds to the void space of Ni and YSZ particles (0.1–1 μm), and the other relates to the effective small pores with a diameter between 1 and 10 μm explicitly introduced by the phase-inversion method. These results agree well with previous FE-SEM observations (Figure 2). The total intruded volume and the porosity of the two different hydrogen electrodes are listed in Table S1. By using the phase-inversion method, the intruded volume in the electrode was increased from 0.067 to 0.24 cm^3^ g^−1^, and the porosity was also increased from 32.1% to 60.8%. In a previous work, Suzuki et al. reported that the porosity of a tubular SOFC was increased from 37% to 54% by tailoring the calcination temperatures, thus achieving an effective improvement in the peak power density from 0.2 to 1 W cm^−2^ [34]. Therefore, the electrode synthesized by the phase-inversion tape-casting method with high porosity is expected to deliver a higher electrochemical performance in SOCs.

**Figure 4:**
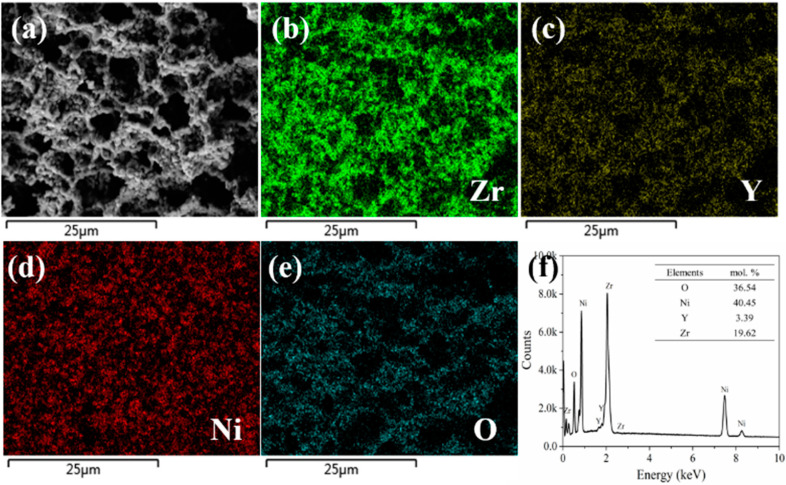
(**a**) STEM image of the cross-section of the phase-inversion tape-casting hydrogen electrode after reduction in H_2_ at 750 °C for 5 h. Elemental mapping of Zr (**b**), Y (**c**), Ni (**d**), and O (**e**), and STEM-EDX result of Ni-YSZ (**f**).

To further prove that the nickel and YSZ in the prepared Ni-YSZ hydrogen electrode by the phase-inversion tape-casting method were evenly distributed, a STEM-EDX analysis was performed for the reduced hydrogen electrode at 750 °C for 5 h, with the typical images shown in Figure 4. There was no apparent aggregation of Ni or YSZ particles, and the Ni and YSZ particles were closely connected, effectively forming the continuous oxygen ion diffusion path (YSZ) and electron conduction path (Ni) (Figure 4b–e). The prepared Ni-YSZ electrode’s elemental composition was further illustrated by the EDX test (Figure 4f), which is consistent with the initial element ratio of Ni and YSZ.

### 2.2. Water Resistance of Oxygen Electrode

**Figure 5:**
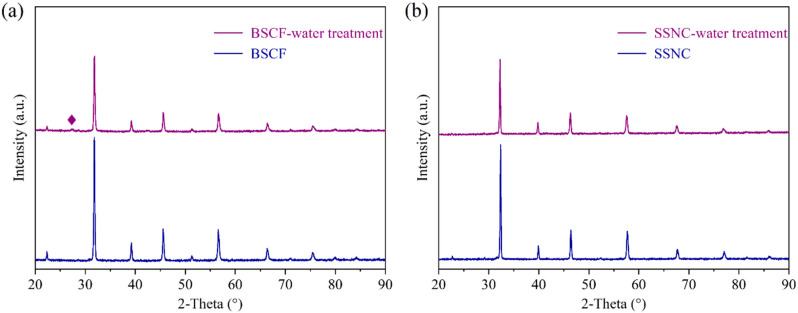
XRD patterns of the oxygen electrodes before and after 10% H_2_O-Ar treatment at 750 °C for 5 h: (**a**) BSCF; (**b**) SSNC. ◆ Ba(OH)_2_ impurity.

It is crucial for SOCs to have high activity concerning oxygen reduction reactions (ORRs) and oxygen evolution reactions (OERs) and prolonged durability of the oxygen electrode when working at low-to-intermediate temperatures. Zhou et al. have confirmed that the perovskite material SrSc_0.175_Nb_0.025_Co_0.8_O_3−δ_ (SSNC) is an excellent candidate because of its relatively high ORR activity and low activation energy, which was enhanced to 100% at 500 °C relative to the benchmark oxygen electrode of BSCF [22]. Zhang et al. also confirmed that SSNC had a higher ORR activity and CO_2_-resistance than BSCF [39]. To further confirm the high activity of SSNC, an O_2_-TPD test was carried out. As shown in Figure S5, during the programmed increase of temperature, the thermal reduction of B-site cations to a lower oxidation state occurs, along with the oxidation of O^2−^ to O_2_, which is finally released into the surrounding atmosphere (via flowing inert gas). It is evident that the peak area of SSNC is much higher than that of BSCF, indicating that SSNC has more variable oxygen vacancies than BSCF [39]. However, in the SOEC mode, because a large amount of water vapor is introduced into the hydrogen electrode, which may be penetrated by the oxygen electrode, and a stable phase structure and micromorphology under an absolute water pressure is vital for oxygen electrodes for SOCs. Figure 5 shows the XRD patterns of the BSCF and SSNC before and after treatment in 10% H_2_O-Ar at 750 °C for 5 h. Obviously, a significant impurity peak appeared at the 2-theta position at about 26° in the BSCF sample (Figure 5a). Yan et al. found the presence of a OH group after the treatment of BSCF in an atmosphere containing H_2_O [40]. Wang et al. also confirmed that Ba(OH)_2_ could be formed on Ba_0.9_Co_0.7_Fe_0.2_Nb_0.1_O_3−δ_ (BCFN) after treatment under an atmosphere containing H_2_O [41]. During the electrolysis process, the presence of Ba(OH)_2_ on the BSCF oxygen electrode’s surface may inhibit the oxygen evolution process. Nevertheless, for the SSNC perovskite, no impurity peaks appeared after treatment in 10% H_2_O-Ar atmosphere, indicating its high resistance to water poisoning (Figure 5b). This is mainly because the alkalinity of the Ba element is greater than that of the Sr element, so water reacts more easily with BSCF to form Ba(OH)_2_. Zhang et al. found that CO_2_ is more likely to poison BSCF due to Ba’s strong alkalinity, while SSNC is much more CO_2_-resistant than BSCF [39].

**Figure 6:**
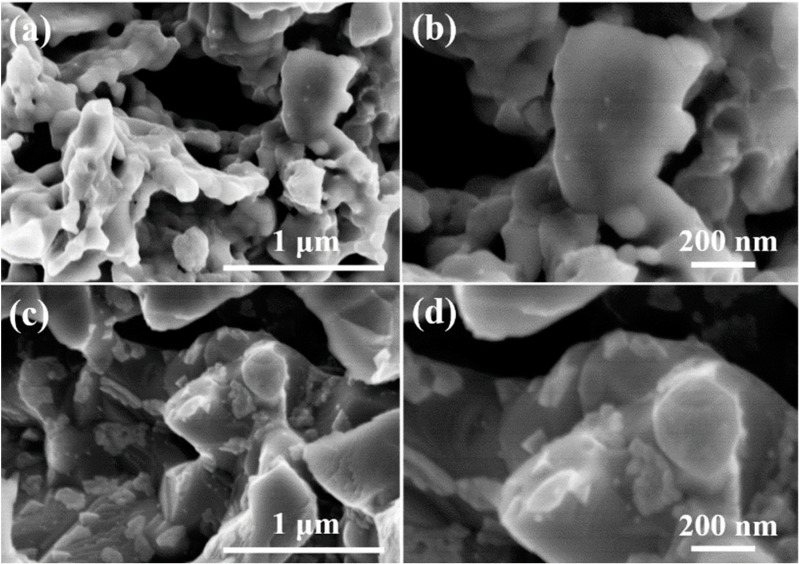
FE-SEM images of the oxygen electrodes after 10% H_2_O-Ar treatment at 750 °C for 5 h: (**a**,**b**) SSNC; (**c**,**d**) BSCF.

To further verify the different effects of H_2_O on the SSNC and BSCF perovskites, the electrode microstructure was examined after the water treatment. In our previous work, the surface roughness of BSCF and SSCN samples before treatment is close to each other with no significant difference, which was illustrated by scanning electron microscopy and atomic force microscopy [39]. As shown in Figure 6a,b, the surface of SSNC perovskite was smooth without the presence of any impurities, while there were large amounts of impurities with a particle size distribution of 100–200 nm emerging on the surface of BSCF perovskite (Figure 6c,d). According to the XRD results, such impurities on the BSCF surface were mainly Ba(OH)_2_. Therefore, in addition to the higher bulk oxygen diffusion rate and oxygen reduction activity than BSCF, SSNC also has a stronger resistance towards water poisoning, making it a more promising oxygen electrode for SOCs compared with BSCF.

### 2.3. SOFC Mode

**Figure 7:**
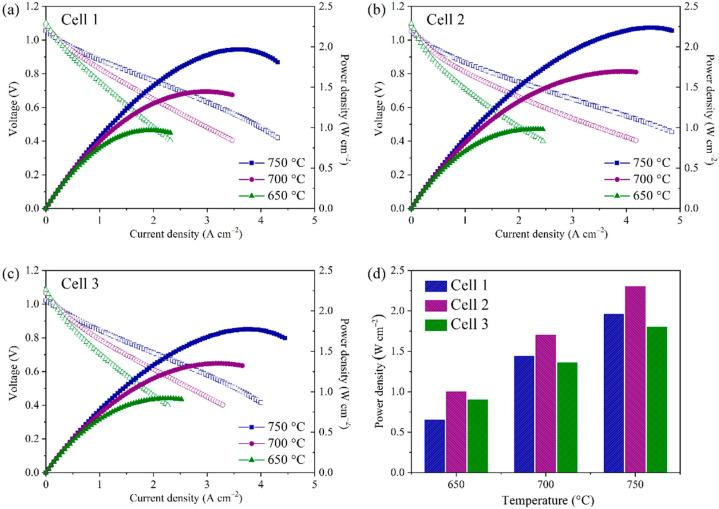
*I-V* and *I-P* curves for the single cells measured at different temperatures. (**a**) Cell 1, (**b**) Cell 2, (**c**) Cell 3, and (**d**) a comparison of peak power densities of three cells at the operating temperatures.

Cell 1 was first tested in the SOFC mode and compared with the other two cells listed in Table 1. Figure 7a–c exhibits the *I-V* and *I-P* curves where three cells with different compositions work at various temperatures of 650–750 °C in the atmosphere consisting of humidified H_2_ as the fuel and ambient air as the oxidant. The open-circuit voltages (OCVs) of Cell 1, Cell 2, and Cell 3 at 750 °C were 1.06, 1.06, and 1.03 V, respectively, which indicated that YSZ electrolyte could be densified and the porous hydrogen electrode prepared by the phase-inversion method did not affect the compactness of the three cells. In comparison, Huang et al. reported a cell with finger-like large pores on the hydrogen electrode, which was prepared via the phase-inversion tape-casting method and showed an OCV of only 1.00 V at 750 °C [29]. Significantly, the peak power densities (PPDs) of the three cells were 1.44, 1.70, 1.36 W cm^−2^ at 700 °C, respectively. Obviously, the power densities of these cells produced by the phase-inversion tape-casting method are superior to those synthesized through the traditional tape-casting method when compared under similar testing conditions. Similarly, the SSNC cathode cells had higher power densities than those that made up of BSCF cathode. The detailed PPDs of the three cells at the specific operating temperatures are shown in Figure 7d, suggesting that the performance in the SOFC mode was enhanced by optimizing the microstructure of the hydrogen electrode and the reactivity of the oxygen electrode. The performances of various single cells containing Ni-based hydrogen electrodes, YSZ electrolyte, and other well-known oxygen electrodes are compared in Table 2 [28,29,37,42,43,44,45,46,47,48,49,50,51]. It was found that the cells consisted of a hydrogen electrode with a honeycomb-like structure, and the oxygen electrode with SSNC cathode demonstrated the best electrochemical performance.

### 2.4. SOEC Mode

**Figure 8:**
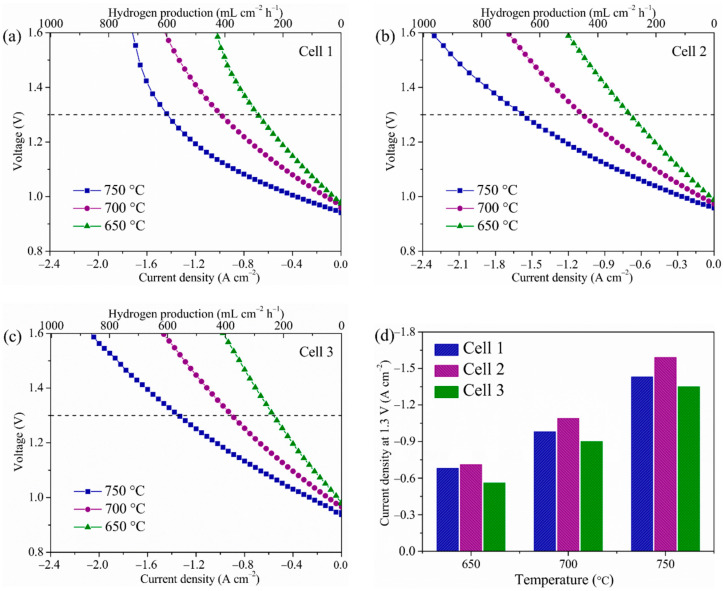
Voltage versus current density measured at different temperatures in air and 50 vol.% H_2_-50 vol.% H_2_O. (**a**) Cell 1, (**b**) Cell 2, (**c**) Cell 3, and (**d**) comparison of the current densities at 1.3 V of the three cells at different temperatures.

Figure 8a–c presents the temperature-dependent *I-V* curves of the three different cells for electrolysis. Within the test period, the volume flow ratio of H_2_O/H_2_ was maintained at 1:1, and the test voltages started from OCVs to −1.6 V with a current sweeping rate of 50 mA s^−1^. Thus, it was necessary to utilize Faraday’s law to calculate the H_2_ production rate at the corresponding current density. As seen in the following, Faraday’s law is ΔN_H2_ = I/2F, where ΔN_H2_ is the H_2_ production rate (mL cm^−2^ h^−1^), and I and F is the current (A) and Faraday’s constant (C), respectively [52,53]. When the thermo-neutral voltage for steam electrolysis is 1.3 V, the current densities of three cells are −1.43, −1.59, and −1.35 A cm^−2^ at 750 °C, and the corresponding derived H_2_ production rate are 597.6, 664.5, and 564.1 mL cm^−2^ h^−1^ at 750 °C, respectively. Meanwhile, it can be seen that Cell 1 showed an obvious polarization phenomenon at 750 °C, which cannot be seen for the other two cells. This is due to the water electrolysis reaction at higher temperatures needing much more reactant with a high concentration of water compared with that of lower temperatures, and the insufficient porosity (32.1%) of Cell 1 cannot satisfy the demands for water diffusion. Therefore, Cell 1 showed an obvious concentration polarization phenomenon at 750 °C. However, the phase-inversion tape-casting hydrogen electrodes had good porosity (60.8%); the concentration polarization phenomenon cannot be seen in the *I-V* curves of Cell 2 and Cell 3. Figure 8d illustrates the detailed current densities of three cells at the operating voltage and temperature. It was found the electrolysis performance of Cell 2 is superior to Cell 1 and Cell 3, implying that the electrolysis performance of cell was successfully strengthened by combining the phase-inversion tape-casted hydrogen electrode with the SSNC oxygen electrode. Moreover, the electrolysis performances of various single cells with the Ni-based hydrogen electrodes, YSZ electrolyte, and other oxygen electrodes are compared in Table 3 [30,44,46,48,49,50,54,55,56,57]. Again, SOECs consisted of the hydrogen electrode with a honeycomb-like structure and the SSNC oxygen electrode show the best electrochemical performance.

To further research the electrochemical performance, it is essential to exert the electrochemical impedance spectra (EIS) to measure the impedance value of the SOECs, and the final results of this investigation are shown in Figure S6a–c. The polarization resistance (R_p_) of Cell 2 is smaller than those of the other two cells under the same conditions (Figure S6d). These results are consistent with the SOC performance. Simultaneously, the three cells’ ohmic resistances (R_o_) are basically the same, indicating that the three cells’ electrolyte thicknesses are almost the same.

### 2.5. Stability

**Figure 9:**
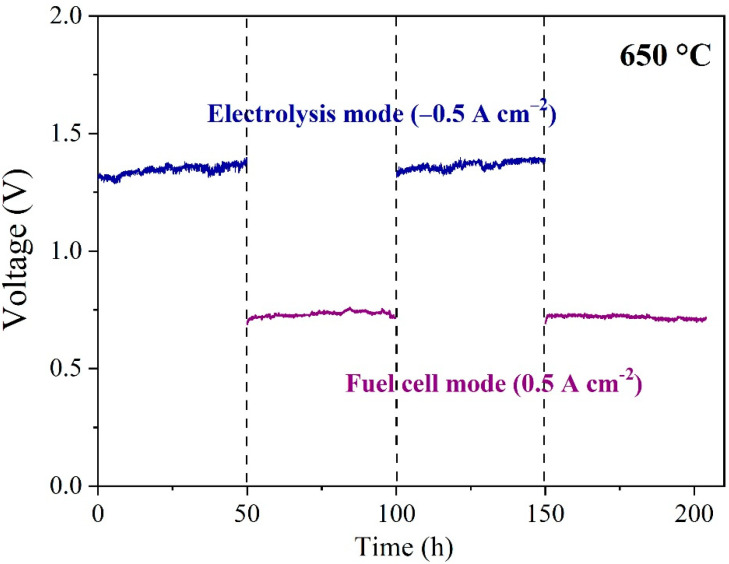
The stability of Cell 2 operated between the electrolysis (@ −0.5 A cm^−2^) and fuel cell modes (@ 0.5 A cm^−2^) at 650 °C.

It is known that the easy agglomeration of Ni nanoparticles at high temperatures is a severe problem during SOEC operation [58,59]. The accumulation of Ni crystal grains at high temperature may be accelerated owing to the relatively low melting point of metallic Ni and the adverse interaction between YSZ and Ni, which means that it could seriously damage the contact surface between the Ni metal and the electrolyte substrate. This is the main reason why it reduced electronic conductivity and declined the area of the three-phase boundary. Accordingly, the cell performance has been expected to deteriorate over a long time, which results from increasing polarization and ohmic impedances. The durability of Cell 2 was tested in both the SOFC and SOEC modes. Figure 9 shows the variation of cell voltage concerning operation time in the alternating operation mode for a period of 50 h under −0.5 A cm^−2^ for the electrolysis mode and then 50 h of operation at 0.5 A cm^−2^ for the fuel cell mode with a total period of 200 h. Although the voltage slightly increased from 1.3 V to 1.39 V during the first 50 h under the electrolysis mode, the voltage returned to 1.31 V when the cell was electrolyzed for the second 50 h. SOCs based on Ni-based hydrogen electrodes can effectively suppress the electrolytic performance by switching the SOEC and SOFC modes. In the SOFC mode, no significant performance degradation was observed in two different sets of 50 h operation. Therefore, the SOCs with the phase-inversion tape-casted hydrogen electrode and SSNC oxygen electrode have a good stability and potential application prospect.

After the stability test, the cross-sectional FE-SEM image of Cell 2 (Ni-YSZ|YSZ|SDC|SSNC) was conducted, as shown in Figure S7, demonstrating that the thickness of the YSZ electrolyte, SDC barrier layer, and SSNC oxygen electrode were ~4.5 μm, ~2 μm, and ~9 μm, respectively. The thin YSZ electrolyte layer was still dense without the appearance of obvious holes. Simultaneously, the SSNC oxygen electrode and electrolyte did not show any delamination after 200 h of testing.

## 3. Materials and Methods

### 3.1. Powder Preparation

SrSc_0.175_Nb_0.025_Co_0.8_O_3−δ_ (SSNC) oxide powder was prepared by a standard solid-state reaction method. In detail, according to the nominal composition of the perovskite oxides, stoichiometric amounts of SrCO_3_, Sc_2_O_3_, Nb_2_O_5_, and Co_3_O_4_ were weighted and mixed with ethanol in a ball milling machine for 30 min. After drying at 180 °C for 5 h, the precursor powder was calcined at 1200 °C for 10 h in air to obtain pure SSNC perovskite power. Using the detailed procedure presented in the literature, a classic EDTA-citric acid complexing sol-gel process was introduced and used to synthesize the oxygen electrode materials, Ba_0.5_Sr_0.5_Co_0.8_Fe_0.2_O_3−δ_ (BSCF) perovskite oxide [32]. Meanwhile, Sm_0.2_Ce_0.8_O_1.9_ (SDC) oxide powder, acting as a buffer layer of the YSZ electrolyte of SOCs in this study, was synthesized by a hydrothermal process [33], where the YSZ was a commercial product purchased from Tosoh, Japan.

### 3.2. Single Cell Preparation

A hydrogen electrode-supported half-cell was synthesized through the phase-inversion tape-casting method. Usually, the single cell consists of the Ni-YSZ cermet hydrogen electrode, the YSZ electrolyte (SDC as a barrier), and the SSNC or BSCF oxygen electrode. The phase-inversion tape-casting method was introduced to create the unreduced NiO-YSZ (NiO and YSZ in a mass ratio of 6:4) hydrogen electrode. In detail, NiO and YSZ oxide powders were mixed well through ball milling at a mass ratio of 6:4, and a proper amount of ethanol and xylene were added during the ball milling process as solvents; fish oil was added as the dispersing agent. The milling was first conducted in a planetary ball mill for 24 h to ensure sufficient dispersion between the NiO and YSZ. Then, a second feeding was performed. An appropriate amount of polyvinyl butyral ester as a binder and dioctyl phthalate and polyethylene glycol 400 as plasticizers was added to the solution, which was ball remixed for another 24 h to obtain a slurry that would be used for subsequent tape-casting. The prepared hydrogen electrode mixture’s slurry was degassed for 5 min by the vacuum pump and casted with a 2 mm height gap. A part of the tape-casting hydrogen electrode was directly dried at room temperature for 24 h to prepare a traditional tape-casting hydrogen electrode, and the other part was directly immersed in water for 24 h to prepare a phase-inversion tape-casting hydrogen electrode. The detailed preparation process for the traditional tape-casting and phase-inversion tape-casting hydrogen electrodes was shown in Figure S1. A hydrogen electrode pellet was formed by punching the tape-casted film using a puncher with a 16 mm diameter. Through a 2 h heating process in air, the punched pellets were fired to 1000 °C at a heating rate of 1 °C min^−1^. The hydrogen electrode surface was sprayed with the prepared YSZ slurry, and the sprayed pellet continued to be calcined at 1400 °C for 5 h in air. Moreover, spraying the SDC slurry on the YSZ electrolyte uniformly and calcining it in air at 1300 °C for 5 h should not be negligible, as the formation of the SDC barrier layer was aimed at avoiding adverse phase reaction happening at the contact surface between the electrolyte and the oxygen electrode. Eventually, in order to fabricate the entire single cell, it was essential to uniformly spray the SSNC oxygen electrode slurry on the surface of the SDC layer, where the effective area was 0.45 cm^2^, and the layer was calcined in air at 900 °C for 2 h. In order to enhance the current collection effect, silver paste was brushed on the oxygen electrode surface. For the convenience of distinguishing different SOCs, the cells were named as follows: Cell 1, composed of a tape-casting hydrogen electrode and SSNC oxygen electrode; Cell 2, composed of a phase-inversion tape-casting hydrogen electrode and SSNC oxygen electrode; and Cell 3, composed of a phase-inversion tape-casting hydrogen electrode and BSCF oxygen electrode. The different structures of the three cells are listed in Table 1.

### 3.3. Characterization and Measurements

To verify the phase structure of the SSNC and BSCF perovskite powders, it was necessary to exert room temperature X-ray diffraction (XRD, D8 Advance, Bruker, Billerica, MA, USA) to characterize them. The microstructural features of the cell components were examined with the use of scanning electron microscopy (FE-SEM, HITACHI-S4800). Silver adhesive was used to seal and fix the prepared cell on the end of a tubular alumina, and silver ink and wires were applied to contact and collect electricity. Electrochemical impedance spectra (EIS) were measured under the frequency range of 0.1 Hz–100 kHz with a signal amplitude of 10 mV in an open-circuit voltage (OCV). When tested in the SOFC mode, the flow rate of pure hydrogen was set to 80 mL min^−1^ [STP] to supply the hydrogen electrode chamber; the oxygen electrode was exposed to the static ambient air, and the cells operated in the temperature range of 750–650 °C. The operating temperature selection is a balance between the performance and degradation factors. In the test of the SOEC mode, hydrogen and water vapor were pre-mixed at a volume ratio of 50:50 and then supplied into the hydrogen electrode chamber, where the oxygen electrode was directly exposed to the ambient air atmosphere. Furthermore, for the stability in both the SOFC and SOEC modes, these cells were tested at a fixed current density of ±0.5 A cm^−2^. Different from the single-cell performance test, in order to ensure that the collector did not sinter at high temperatures, a silver mesh was used as the single-cell collector for stability testing instead of silver paste, sacrificing part of the cell’s performance.

## 4. Conclusions

In this paper, solid oxide cells with a phase-inversion tape-casted hydrogen electrode and SrSc_0.175_Nb_0.025_Co_0.8_O_3−δ_ oxygen electrode were successfully fabricated and investigated. The ordered honeycomb-like structure of the hydrogen electrode was formed through the phase-inversion method, which was favorable to fuel transfer and byproduct generation in SOCs. Compared with the benchmark BSCF oxygen electrode, the SSNC electrode had a stronger resistance to water poisoning. Combined with the phase-inversion tape-casted hydrogen electrode and SSNC oxygen electrode, the SOC achieved a peak power density of 2.3 W cm^−2^ in the SOFC mode and a high current density of −1.59 A cm^−2^ (at 1.3 V) in the SOEC mode at 750 °C. These results demonstrate that the SOC with a SrSc_0.175_Nb_0.025_Co_0.8_O_3−δ_ oxygen electrode and phase-inversion tape-casted hydrogen electrode has promise in water splitting prospects at high temperatures.

## Figures and Tables

**Table 1:** The various parameters of SOCs in this study.

	Hydrogen Electrode	Electrolyte	Oxygen Electrode
Cell 1	Traditional tape casting	YSZ-SDC	SSNC
Cell 2	Phase-inversion tape-casting	YSZ-SDC	SSNC
Cell 3	Phase-inversion tape-casting	YSZ-SDC	BSCF

**Table 2:** Comparison of the electrochemical performance of various SOFCs.

Cell Composition	Temp. (°C)	PPDs (W cm^−2^)	Ref.
Ni-YSZ ^a^|YSZ|SDC|SSNC	750	2.3	This work
Ni-YSZ ^a^|Ni-ScSZ ^b^|ScSZ|LSM-ScSZ|LSM	750	1.32	[28]
Ni-YSZ ^a^|YSZ|LSM-YSZ	800	0.78	[29]
Ni-YSZ-GDC ^c^|GDC|YSZ|GDC|STFC ^d^	750	~1.9	[37]
Ni-YSZ|YSZ|SDC|SC-SDC ^e^	750	~1.75	[42]
Ni-YSZ|YSZ|GDC|BSCF	800	1.56	[43]
Ni-YSZ|YSZ|NBCF ^f^-YSZ	800	1.44	[44]
Ni-YSZ|Ni-ScSZ|ScSZ|MCCO ^g^-ScSZ	800	1.92	[45]
Ni-YSZ|YSZ|GDC|NBCCF ^h^-GDC	850	1.39	[46]
Ni-YSZ|YSZ|SDC|BC-PBCC ^i^	750	~1.15	[47]
Ni-YSZ|YSZ|GDC|PBSCF ^j^	800	~1.0	[48]
Ni-YSZ|YSZ|GDC|GDC-LSCF|LSCF	800	~0.8	[49]
Ni-YSZ|YSZ|SDC|SDC-BNF ^k^	800	1.2	[50]
Ni-YSZ|YSZ|GDC|PCFC ^l^	750	~0.85	[51]

^a^ phase-inversion tape-casting method, ^b^ ScSZ: 10 mol% Sc_2_O_3_-ZrO_2_,^c^ GDC: Ce_0.9_Gd_0.1_O_2_, ^d^ STFC: Sr(Ti_0.3_Fe_0.63_Co_0.07_)O_3−δ_, ^e^ SC-SDC: SrCoO_3−δ_ impregnated into SDC, ^f^ NBCF: NdBaCoFeO_5+δ_, ^g^ MCCO: Mn_1.3_Co_1.3_Cu_0.4_O_4_, ^h^ NBCCF: NdBa_0.5_Ca_0.5_Co_1.5_Fe_0.5_O_5+δ_, ^i^ BC-PBCC: BaCoO_3−δ_-PrBa_0.8_Ca_0.2_Co_2_O_5+δ_, ^j^ PBSCF: PrBa_0.5_Sr_0.5_Co_1.5_Fe_0.5_O_5+δ_, ^k^ BNF: Ba_0.97_Nd_0.03_FeO_3−δ_, ^l^ PCFC: Pr_0.8_Ca_0.2_Fe_0.8_Co_0.2_O_3−δ._

**Table 3:** Comparison of the electrochemical performance of various SOECs for H_2_O electrolysis.

Cell Composition	Temp. (°C)	Gas Composition	j (A cm^−2^) at 1.3 V	Ref.
Ni-YSZ|YSZ|SDC|SSNC	750	H_2_/H_2_O (50/50)	−1.59	This work
Ni-YSZ ^a^|YSZ|LSM-YSZ	800	H_2_/H_2_O (67/33)	−1.35	[30]
Ni-YSZ|YSZ|GDC|PBSCF	750	H_2_/H_2_O (70/30)	−0.6	[44]
Ni-YSZ|YSZ|GDC|NBCCF-GDC	800	H_2_/H_2_O (50/50)	−0.92	[46]
Ni-YSZ|YSZ|GDC|PBSCF	800	H_2_/H_2_O (50/50)	−1.3	[48]
Ni-YSZ|YSZ|GDC|GDC-LSCF|LSCF	800	H_2_/H_2_O (50/50)	−0.85	[49]
Ni-YSZ|YSZ|SDC|SDC-BNF	800	H_2_/H_2_O (50/50)	−2.05	[50]
Ni-YSZ|YSZ|GDC|PCFC	750	H_2_/H_2_O (50/50)	−0.79	[51]
Ni-YSZ|YSZ|SDC|BSCF-SDC	800	H_2_/H_2_O (50/50)	−0.62	[54]
Ni-YSZ|ScSZ|NNO ^b^-ScSZ	800	H_2_/H_2_O (50/50)	−1.08	[55]
Ni-YSZ|YSZ|SDC|PNO ^c^-GDC	800	H_2_/H_2_O (50/50)	−0.78	[56]
NiYSZ|YSZ|SDC|LSFCN ^d^	800	H_2_/H_2_O (50/50)	−1.04	[57]

^a^ phase-inversion tape-casting method, ^b^ NNO: Nd_2_NiO_4+δ_, ^c^ PNO: Pr_2_NiO_4+δ_, ^d^ LSCFN: La_0.5_Sr_0.5_Fe_0.8_Cu_0.15_Nb_0.05_O_3−δ._

## Data Availability

Not applicable.

## Supplementary Materials

The following are available online at https://www.mdpi.com/article/10.3390/molecules27238396/s1. Figure S1: Schematic of the preparation processes for the tape-casting and phase-inversion tape-casting hydrogen electrodes; Figure S2: TG-DTA curves (a) and the MS analysis (b) for the tape-casting and phase-inversion tape-casting hydrogen electrodes before calcined. *m*/*z* = 18 for the H_2_O MS signal and *m*/*z* = 44 for the CO_2_ MS signal; Figure S3: SEM images of the surface of the tape-casting (a,b) and phase-inversion tape-casting (c,d) hydrogen electrodes calcined at 1400 °C for 5 h; Figure S4: Images of the tape-casting (a) and phase-inversion tape-casting (b) hydrogen electrodes calcined at 1400 °C for 5 h in 3 s when the water was dropped onto the surfaces; Figure S5: O_2_-TPD profiles of SSNC and BSCF powders in pure Ar atmosphere from 50 to 800 °C. The inset is the relative area of the SSNC and BSCF; Figure S6: Electrochemical impedance spectra of three cells at OCV at different temperatures with 50% H_2_O–50% H_2_. (a) Cell 1, (b) Cell 2, (c) Cell 3, (d) the polarization resistance and ohmic resistance of the three cells at different temperatures; Figure S7: Cross-section FE-SEM image of Cell 2 after the stability test. Table S1: Parameters of direct tape-casting and phase-inversion tape-casting hydrogen electrodes conducted by mercury intrusion.

## Sample Availability

Samples of the compounds are not available from the authors.

## References

[1] Li C., Zhu D., Cheng S., Zuo Y., Wang Y., Ma C., Dong H. (2022). Recent research progress of bimetallic phosphides-based nanomaterials as cocatalyst for photocatalytic hydrogen evolution. Chin. Chem. Lett..

[2] Yang M., He F., Zhou C., Dong F., Yang G., Zhou W., Shao Z. (2021). New perovskite membrane with improved sintering and self-reconstructed surface for efficient hydrogen permeation. J. Membr. Sci..

[3] Parra D., Valverde L., Pino F.J., Patela M.K. (2019). A review on the role, cost and value of hydrogen energy systems for deep decarbonisation. Renew. Sustain. Energy Rev..

[4] Liu Z., Chen Y., Yang G., Yang M., Ji R., Song Y., Ran R., Zhou W., Shao Z. (2022). One-pot derived thermodynamically quasi-stable triple conducting nanocomposite as robust bifunctional air electrode for reversible protonic ceramic cells. Appl. Catal. B Environ..

[5] Bi L., Boulfrad S., Traversa E. (2014). Steam electrolysis by solid oxide electrolysis cells (SOECs) with proton-conducting oxides. Chem. Soc. Rev..

[6] Lei L., Zhang J., Yuan Z., Liu J., Ni M., Chen F. (2019). Progress report on proton conducting solid oxide electrolysis cells. Adv. Funct. Mater..

[7] Kim S., Kim G., Manthiram A. (2021). Dysprosium doping effects on perovskite oxides for air and fuel electrodes of solid oxide cells. J. Power Sources.

[8] Liu Z., Cheng D., Zhu Y., Liang M., Yang M., Yang G., Ran R., Wang W., Zhou W., Shao Z. (2022). Robust bifunctional phosphorus-doped perovskite oxygen electrode for reversible proton ceramic electrochemical cells. Chem. Eng. J..

[9] Zhang W., Zhou Y., Liu E., Ding Y., Luo Z., Li T., Kane N., Zhao B., Niu Y., Liu Y. (2021). A highly efficient and durable air electrode for intermediate-temperature reversible solid oxide cells. Appl. Catal. B Environ..

[10] Liu Y., Shao Z., Mori T. (2021). Development of nickel based cermet anode materials in solid oxide fuel cells–Now and future. Mater. Rep. Energy.

[11] Yang G., Su C., Shi H., Zhu Y., Song Y., Zhou W., Shao Z. (2020). Toward reducing the operation temperature of solid oxide fuel cells: Our past 15 years of efforts in cathode development. Energy Fuels.

[12] Xu X., Su C., Shao Z. (2021). Fundamental understanding and application of Ba_0.5_Sr_0.5_Co_0.8_Fe_0.2_O_3−δ_ perovskite in energy storage and conversion: Past, present, and future. Energy Fuels.

[13] Liang M., Zhu Y., Song Y., Guan D., Luo Z., Yang G., Jiang S.P., Zhou W., Ran R., Shao Z. (2021). A new durable surface nanoparticles-modified perovskite cathode for protonic ceramic fuel cells from selective cation exsolution under oxidizing atmosphere. Adv. Mater..

[14] Yang T., Kollasch S.L., Grimes J., Xue A., Barnett S.A. (2022). (La_0.8_Sr_0.2_)_0.98_MnO_3−δ_-Zr_0.92_Y_0.16_O_2−δ_: PrO_x_ for oxygen electrode supported solid oxide cells. Appl. Catal. B Environ..

[15] Tong X., Ovtar S., Brodersen K., Hendriksen P.V., Chen M. (2020). Large-area solid oxide cells with La_0.6_Sr_0.4_CoO_3−δ_ infiltrated oxygen electrodes for electricity generation and hydrogen production. J. Power Sources.

[16] Teng Z., Xiao Z., Yang G., Guo L., Yang X., Ran R., Wang W., Zhou W., Shao Z. (2020). Efficient water splitting through solid oxide electrolysis cells with a new hydrogen electrode derived from A-site cation-deficient La_0.4_Sr_0.55_Co_0.2_Fe_0.6_Nb_0.2_O_3−δ_ perovskite. Mater. Today Energy.

[17] Wei B., Chen K., Zhao L., Lü Z. (2015). Chromium deposition and poisoning at La_0.6_Sr_0.4_Co_0.2_Fe_0.8_O_3−δ_ oxygen electrodes of solid oxide electrolysis cells. Phys. Chem. Chem. Phys..

[18] Shao Z., Haile S.M. (2004). A high-performance cathode for the next generation of solid-oxide fuel cells. Nature.

[19] Zhang W., Yu B., Xu J. (2012). Investigation of single SOEC with BSCF anode and SDC barrier layer. Int. J. Hydrogen Energy.

[20] Duan C., Hook D., Chen Y., Tong J., O’Hayre R. (2017). Zr and Y co-doped perovskite as a stable, high performance cathode for solid oxide fuel cells operating below 500 °C. Energy Environ. Sci..

[21] Kuai X., Yang G., Chen Y., Sun H., Dai J., Song Y., Ran R., Wang W., Zhou W., Shao Z. (2019). Boosting the activity of BaCo_0.4_Fe_0.4_Zr_0.1_Y_0.1_O_3−δ_ perovskite for oxygen reduction reactions at low-to-intermediate temperatures through tuning B-site cation deficiency. Adv. Energy Mater..

[22] Zhou W., Sunarso J., Zhao M., Liang F., Klande T., Feldhoff A. (2013). A highly active perovskite electrode for the oxygen reduction reaction below 600 °C. Angew. Chem. Int. Ed..

[23] Trini M., Hauch A., De Angelis S., Tong X., Hendriksen P.V., Chen M. (2020). Comparison of microstructural evolution of fuel electrodes in solid oxide fuel cells and electrolysis cells. J. Power Sources.

[24] Wang Y., Lei X., Zhang Y., Chen F., Liu T. (2018). In-situ growth of metallic nanoparticles on perovskite parent as a hydrogen electrode for solid oxide cells. J. Power Sources.

[25] Yoon K.J., Lee S.I., An H., Kim J., Son J.W., Lee J.H., Je H.J., Lee H.W., Kim B.K. (2014). Gas transport in hydrogen electrode of solid oxide regenerative fuel cells for power generation and hydrogen production. Int. J. Hydrogen Energy.

[26] Kim J., Jun A., Gwon O., Yoo S., Liu M., Shin J., Lim T.H., Kim G. (2018). Hybrid-solid oxide electrolysis cell: A new strategy for efficient hydrogen production. Nano Energy.

[27] Chen T., Zhou Y., Liu M., Yuan C., Ye X., Zhan Z., Wang S. (2015). High performance solid oxide electrolysis cell with impregnated electrodes. Electrochem. Commun..

[28] Sun W., Zhang N., Mao Y., Sun K. (2012). Preparation of dual-pore anode supported Sc_2_O_3_-stabilized-ZrO_2_ electrolyte planar solid oxide fuel cell by phase-inversion and dip-coating. J. Power Sources.

[29] Huang H., Lin J., Wang Y., Wang S., Xia C., Chen C. (2015). Facile one-step forming of NiO and yttrium-stabilized zirconia composite anodes with straight open pores for planar solid oxide fuel cell using phase-inversion tape casting method. J. Power Sources.

[30] Liu T., Wang Y., Zhang Y., Fang S., Lei L., Ren C., Chen F. (2015). Steam electrolysis in a solid oxide electrolysis cell fabricated by the phase-inversion tape casting method. Electrochem. Commun..

[31] Gao J., Meng Y., Hong T., Kim S., Lee S., He K., Brinkman K.S. (2019). Rational anode design for protonic ceramic fuel cells by a one-step phase inversion method. J. Power Sources.

[32] Gu H., Yang G., Hu Y., Liang M., Chen S., Ran R., Xu M., Wang W., Zhou W., Shao Z. (2020). Enhancing the oxygen reduction activity of PrBaCo_2_O_5+δ_ double perovskite cathode by tailoring the calcination temperatures. Int. J. Hydrogen Energy.

[33] Shi H., Zhou W., Ran R., Shao Z. (2010). Comparative study of doped ceria thin-film electrolytes prepared by wet powder spraying with powder synthesized via two techniques. J. Power Sources.

[34] Suzuki T., Hasan Z., Funahashi Y., Yamaguchi T., Fujishiro Y., Awano M. (2009). Impact of anode microstructure on solid oxide fuel cells. Science.

[35] Zheng K., Ni M. (2016). Reconstruction of solid oxide fuel cell electrode microstructure and analysis of its effective conductivity. Sci. Bull..

[36] Jin C., Yang C., Chen F. (2010). Effects on microstructure of NiO–YSZ anode support fabricated by phase-inversion method. J. Membr. Sci..

[37] Park B.K., Scipioni R., Barnett S.A. (2020). Enhancement of Ni–(Y_2_O_3_)_0.08_(ZrO_2_)_0.92_ fuel electrode performance by infiltration of Ce_0.8_Gd_0.2_O_2−δ_ nanoparticles. J. Mater. Chem. A.

[38] Qiao J., Zhang N., Wang Z., Mao Y., Sun K., Yuan Y. (2009). Performance of mix-impregnated CeO_2_-Ni/YSZ anodes for direct oxidation of methane in solid oxide fuel cells. Fuel Cells.

[39] Zhang Y., Yang G., Chen G., Ran R., Zhou W., Shao Z. (2016). Evaluation of the CO_2_ poisoning effect on a highly active cathode SrSc_0.175_Nb_0.025_Co_0.8_O_3−δ_ in the oxygen reduction reaction. ACS Appl. Mater. Interfaces.

[40] Yan A., Liu B., Dong Y., Tian Z., Wang D., Cheng M. (2008). A temperature programmed desorption investigation on the interaction of Ba_0.5_Sr_0.5_Co_0.8_Fe_0.2_O_3−δ_ perovskite oxides with CO_2_ in the absence and presence of H_2_O and O_2_. Appl. Catal. B Environ..

[41] Wang J., Yang Z., Ba L., Chen Y., Ge B., Peng S. (2018). Effects of CO_2_ and H_2_O on Ba_0.9_Co_0.7_Fe_0.2_Nb_0.1_O_3−δ_ cathode and modification by a Ce_0.9_Gd_0.1_O_2−δ_ coating. J. Electroanal. Chem..

[42] Chen D., Yang G., Ciucci F., Tadé M.O., Shao Z. (2014). 3D core–shell architecture from infiltration and beneficial reactive sintering as highly efficient and thermally stable oxygen reduction electrode. J. Mater. Chem. A..

[43] Duan Z., Yang M., Yan A., Hou Z., Dong Y., Chong Y., Chen M., Yang W. (2006). Ba_0.5_Sr_0.5_Co_0.8_Fe_0.2_O_3−δ_ as a cathode for IT-SOFCs with a GDC interlayer. J. Power Sources.

[44] Yoo S., Shin J.Y., Kim G. (2011). Thermodynamic and electrical properties of layered perovskite NdBaCo_2−x_Fe_x_O_5+δ_−YSZ (x = 0, 1) composites for intermediate temperature SOFC cathodes. J. Electrochem. Soc..

[45] Thaheem I., Kim K.J., Lee J.J., Joh D.W., Jeong I., Lee K.T. (2019). High performance Mn_1.3_Co_1.3_Cu_0.4_O_4_ spinel based composite cathodes for intermediate temperature solid oxide fuel cells. J. Mater.Chem. A.

[46] Tian Y., Liu Y., Wang W., Jia L., Pu J., Chi B., Li J. (2020). High performance and stability of double perovskite-type oxide NdBa_0.5_Ca_0.5_Co_1.5_Fe_0.5_O_5+δ_ as an oxygen electrode for reversible solid oxide electrochemical cell. J. Energy Chem..

[47] Chen Y., Yoo S., Zhang W., Kim J.H., Zhou Y., Pei K., Kane N., Zhao B., Murphy R., Choi Y.M. (2019). Effective promotion of oxygen reduction reaction by in situ formation of nanostructured catalyst. ACS Catal..

[48] Tian Y., Li J., Liu Y., Yang J., Liu B., Jia L., Jiang J., Chi B., Pu J., Li J. (2018). Preparation and properties of PrBa_0.5_Sr_0.5_Co_1.5_Fe_0.5_O_5+δ_ as novel oxygen electrode for reversible solid oxide electrochemical cell. Int. J. Hydrogen Energy.

[49] López-Robledo M.J., Laguna-Bercero M.A., Larrea A., Orera V.M. (2018). Reversible operation of microtubular solid oxide cells using La_0.6_Sr_0.4_Co_0.2_Fe_0.8_O_3−δ_-Ce_0.9_Gd_0.1_O_2−δ_ oxygen electrodes. J. Power Sources.

[50] Kim Y.D., Yang J.Y., Saqib M., Park K., Shin J.S., Jo M., Park K.M., Lim H.T., Song S.J., Park J.Y. (2021). Cobalt-free perovskite Ba_1−x_Nd_x_FeO_3−δ_ air electrode materials for reversible solid oxide cells. Ceram. Int..

[51] Li Y., Tian Y., Li J., Pu J., Chi B. (2022). Sr-free orthorhombic perovskite Pr_0.8_Ca_0.2_Fe_0.8_Co_0.2_O_3−δ_ as a high-performance air electrode for reversible solid oxide cell. J. Power Sources.

[52] Yun B.H., Kim K.J., Joh D.W., Chae M.S., Lee J.J., Kim D.W., Kang D., Choi D., Hong S.t., Lee K.T. (2019). Highly active and durable double-doped bismuth oxide-based oxygen electrodes for reversible solid oxide cells at reduced temperatures. J. Mater. Chem. A.

[53] Khan M.S., Xu X., Knibbe R., Zhu Z. (2021). Air electrodes and related degradation mechanisms in solid oxide electrolysis and reversible solid oxide cells. Renew. Sustain. Energy Rev..

[54] Heidari D., Javadpour S., Chan S.H. (2017). Optimization of BSCF-SDC composite air electrode for intermediate temperature solid oxide electrolyzer cell. Energy Convers. Manag..

[55] Chen T., Liu M., Yuan C., Zhou Y., Ye X., Zhan Z., Xia C., Wang S. (2015). High performance of intermediate temperature solid oxide electrolysis cells using Nd_2_NiO_4+δ_ impregnated scandia stabilized zirconia oxygen electrode. J. Power Sources.

[56] Laguna-Bercero M.A., Monzón H., Larrea A., Orera V.M. (2016). Improved stability of reversible solid oxide cells with a nickelate-based oxygen electrode. J. Mater. Chem. A.

[57] Zhou N., Yin Y.M., Li J., Xu L., Ma Z.F. (2017). A robust high performance cobalt-free oxygen electrode La_0.5_Sr_0.5_Fe_0.8_Cu_0.15_Nb_0.05_O_3−δ_ for reversible solid oxide electrochemical cell. J. Power Sources.

[58] Matsui T., Kishida R., Kim J.Y., Muroyama H., Eguchi K. (2000). Performance deterioration of Ni–YSZ anode induced by electrochemically generated steam in solid oxide fuel cells. J. Electrochem. Soc..

[59] Hubert M., Laurencin J., Cloetens P., Morel B., Montinaro D., Lefebvre-Joud F. (2018). Impact of nickel agglomeration on solid oxide cell operated in fuel cell and electrolysis modes. J. Power Sources.

