# Supplementary material for: Solid Oxide Cells with Phase-Inversion Tape-Casted Hydrogen Electrode and SrSc0.175Nb0.025Co0.8O3−δ Oxygen Electrode for High-Performance Reversible Power Generation and Hydrogen Production
Source: Molecules. 2022 Dec 1;27(23):8396. doi: 10.3390/molecules27238396 (PMC9735547; doi:10.3390/molecules27238396)
Supplement: Supplementary file 1 [file molecules-27-08396-s001.zip › molecules-1987853-supplementary.pdf]

Supporting Information

Solid Oxide Cells with Phase-Inversion Tape-Casted Hydrogen Electrode and $SrSc_{0.175}Nb_{0.025}Co_{0.8}O_{3-\delta}$ Oxygen Electrode for High-Performance Reversible Power Generation and Hydrogen Production

Meiting Yang [1], Changjiang Yang [1], Mingzhuang Liang [1], Guangming Yang [1,*], Ran Ran [1], Wei Zhou [1] and Zongping Shao [1,2]

[1] State Key Laboratory of Materials-Oriented Chemical Engineering, College of Chemical Engineering, Nanjing Tech University, Nanjing 211816, China
[2] WA School of Mines: Minerals, Energy and Chemical Engineering, Curtin University, Perth, WA 6845, Australia
* Correspondence: ygm89525@njtech.edu.cn

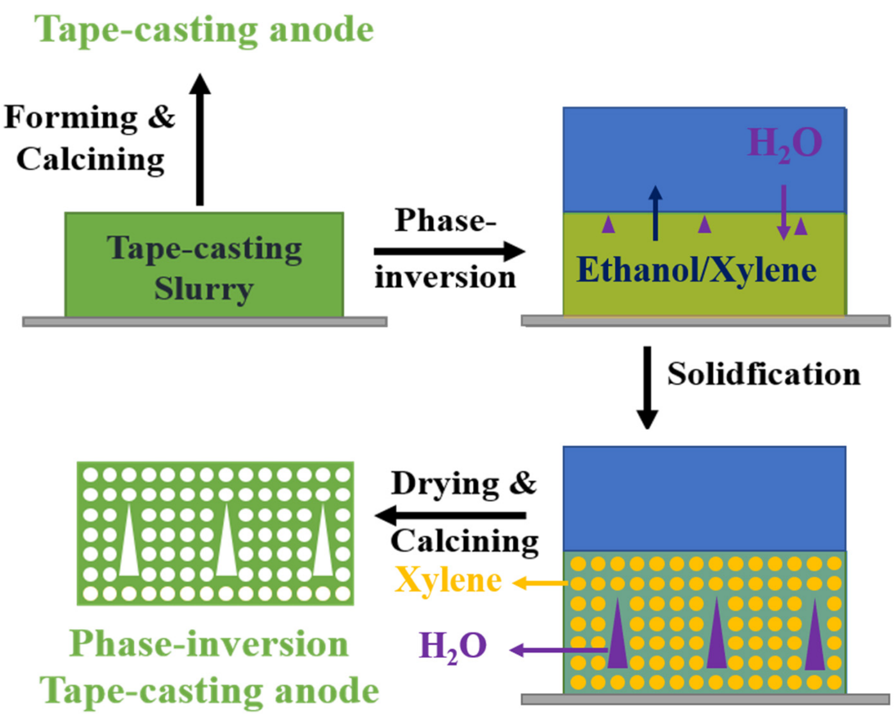

Figure S1 Schematic of the preparation processes for tape-casting and phase-inversion tape-casting hydrogen electrodes.

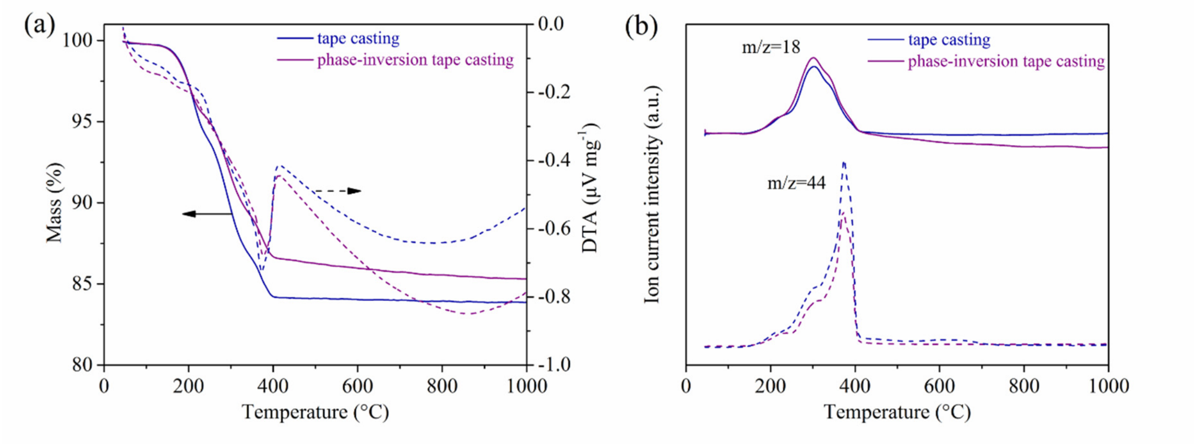

Figure S2 TG-DTA curves (a), and the MS analysis (b) for tape casting and phase-inversion tape casting hydrogen electrode before calcined. m/z = 18 for the H_2O MS signal and m/z = 44 for the CO_2 MS signal.

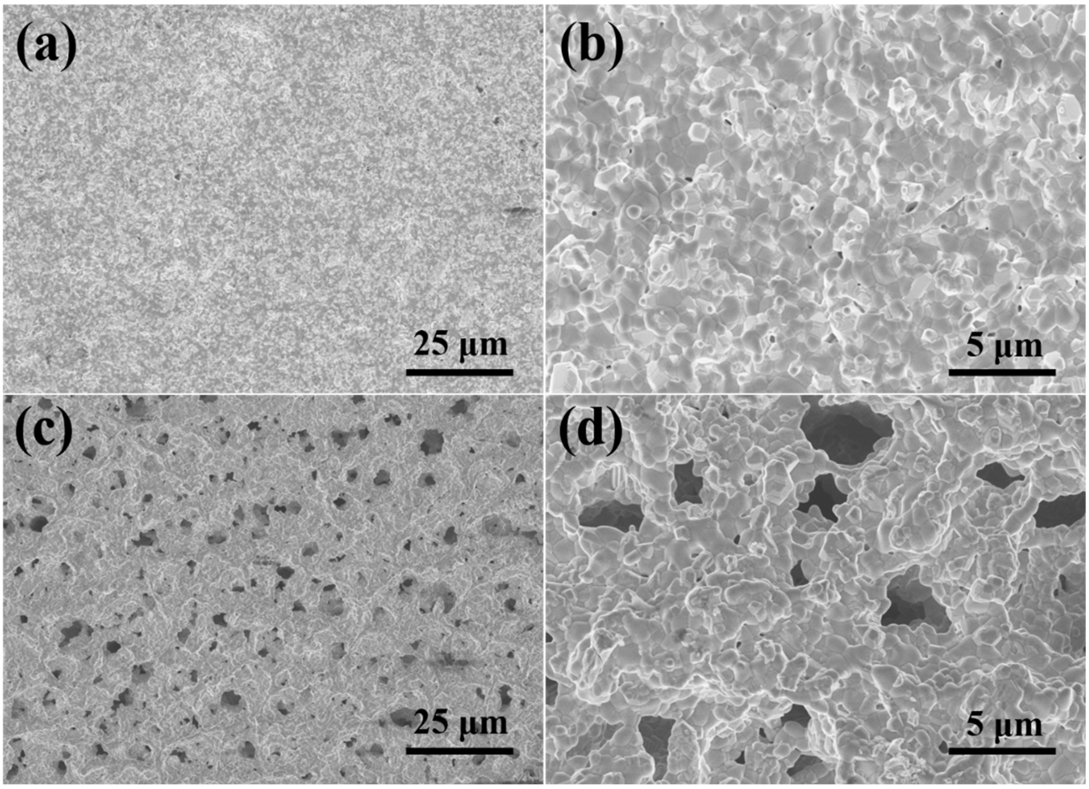

Figure S3 SEM images of the surface of tape-casting (a, b) and phase-inversion tape-casting (c, d) hydrogen electrodes calcined at 1400 °C for 5 h.

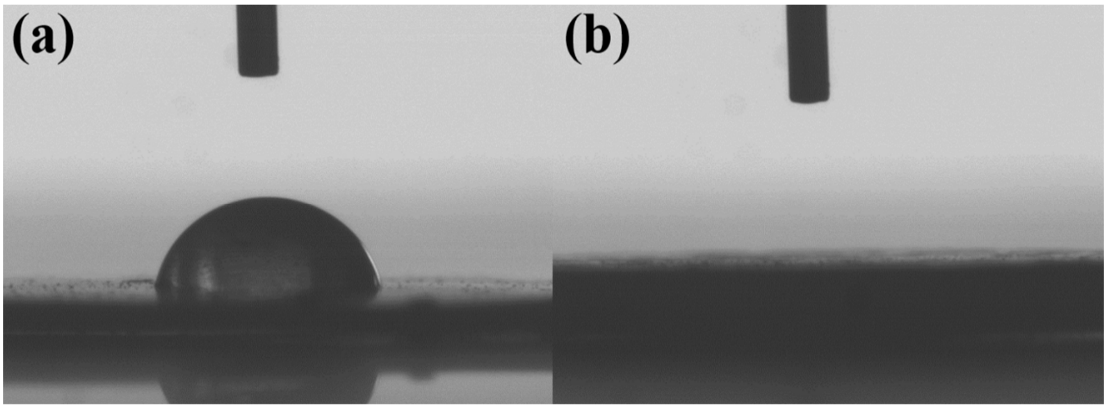

Figure S4 Images of tape-casting (a) and phase-inversion tape-casting (b) hydrogen electrodes calcined at 1400 °C for 5 h in 3 seconds when the water dropped onto the surfaces.

Table S1 Parameters of direct tape casting and phase-inversion tape casting hydrogen electrodes conducted by mercury intrusion.

Sample	Intruded volume ($cm^3\ g^{-1}$)	Bulk density ($g\ cm^{-3}$)	Surface area ($m^2\ g^{-1}$)	Apparent density	Porosity (%)
Tape casting electrode	0.067	4.82	0.96	6.93	32.1
Phase-inversion Tape casting electrode	0.24	2.50	0.82	5.93	60.8

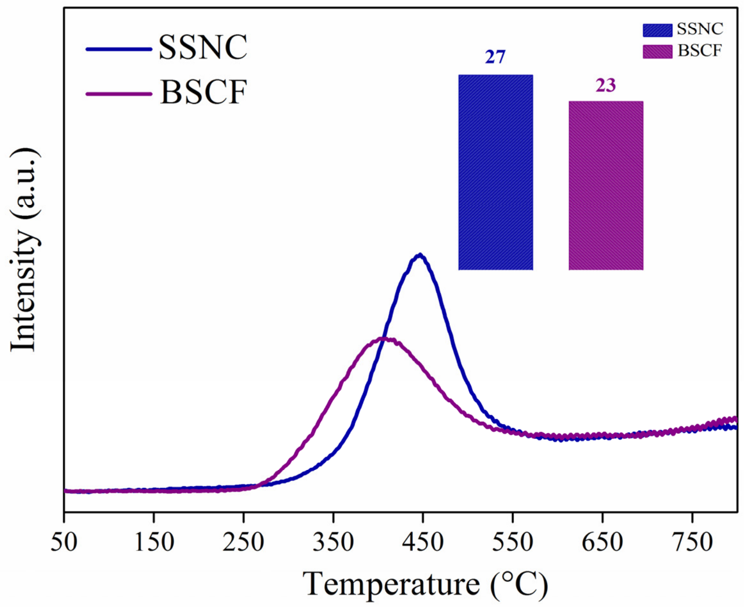

Figure S5 O_2-TPD profiles of SSNC and BSCF powders in pure Ar atmosphere from 50 to 800 °C. The inset is the relative area of SSNC and BSCF.

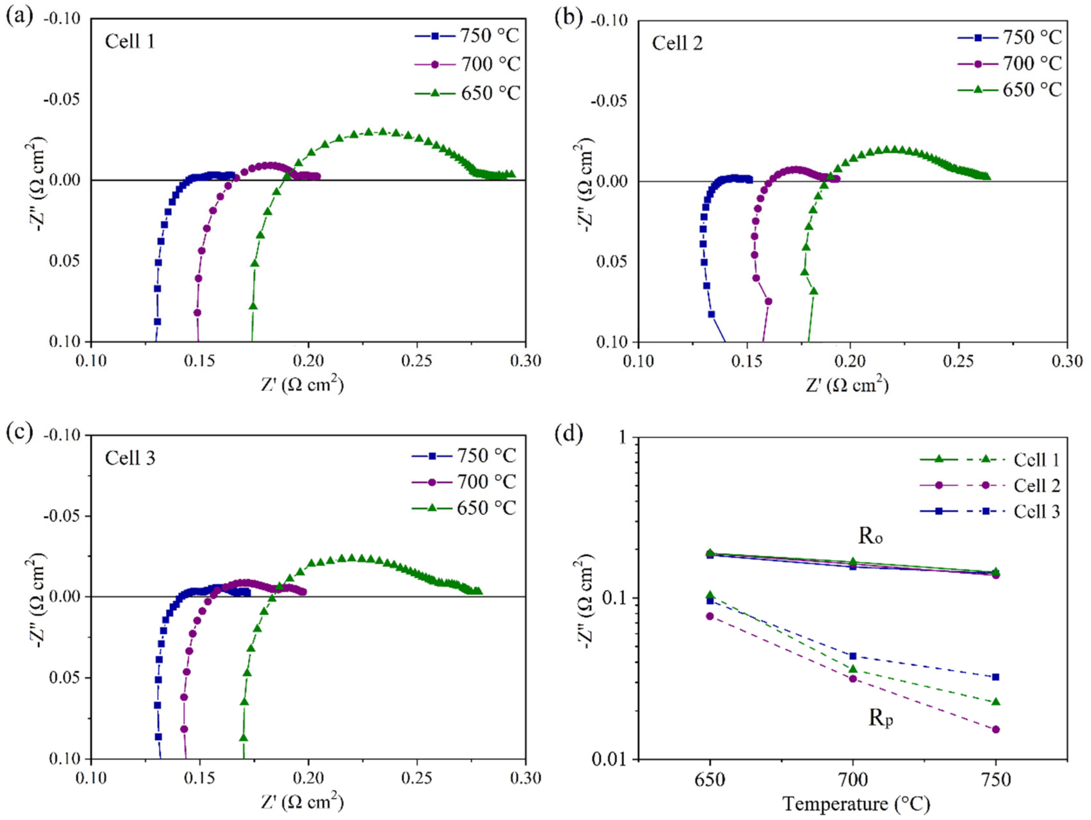

Figure S6 Electrochemical impedance spectra of three cells at OCV at different temperatures. (a) Cell 1, (b) Cell 2, (c) Cell 3, (d) the polarization resistance and ohmic resistance of the three cells at different temperatures.

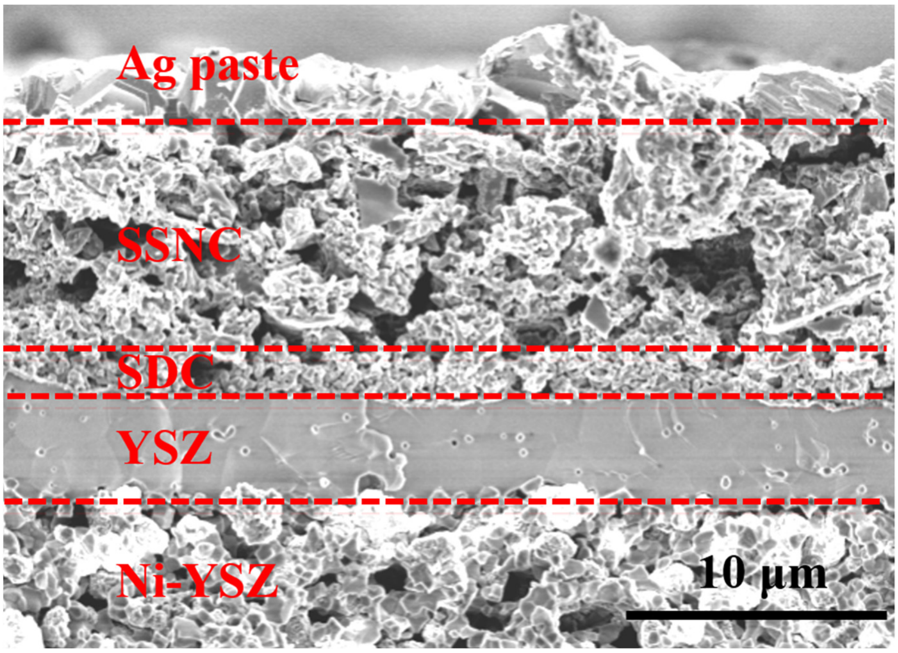

Figure S7 Cross-section FE-SEM image of the Cell 2 after the stability test
